# Microbiota instruct IL-17A-producing innate lymphoid cells to promote skin inflammation in cutaneous leishmaniasis

**DOI:** 10.1371/journal.ppat.1009693

**Published:** 2021-10-26

**Authors:** Tej Pratap Singh, Augusto M. Carvalho, Laís Amorim Sacramento, Elizabeth A. Grice, Phillip Scott

**Affiliations:** 1 Department of Pathobiology, University of Pennsylvania, Philadelphia, Pennsylvania, United States of America; 2 Department of Dermatology, Perelman School of Medicine, University of Pennsylvania, Philadelphia, Pennsylvania, United States of America; INRS - Institut Armand Frappier, CANADA

## Abstract

Innate lymphoid cells (ILCs) comprise a heterogeneous population of immune cells that maintain barrier function and can initiate a protective or pathological immune response upon infection. Here we show the involvement of IL-17A-producing ILCs in microbiota-driven immunopathology in cutaneous leishmaniasis. IL-17A-producing ILCs were RORγt^+^ and were enriched in *Leishmania major* infected skin, and topical colonization with *Staphylococcus epidermidis* before *L*. *major* infection exacerbated the skin inflammatory responses and IL-17A-producing RORγt^+^ ILC accumulation without impacting type 1 immune responses. IL-17A responses in ILCs were directed by *Batf3* dependent CD103^+^ dendritic cells and IL-23. Moreover, experiments using *Rag1*^*-/-*^ mice established that IL-17A^+^ ILCs were sufficient in driving the inflammatory responses as depletion of ILCs or neutralization of IL-17A diminished the microbiota mediated immunopathology. Taken together, this study indicates that the skin microbiota promotes RORγt^+^ IL-17A-producing ILCs, which augment the skin inflammation in cutaneous leishmaniasis.

## Author summary

Cutaneous leishmaniasis includes a spectrum of diseases ranging from a single ulcerative lesion to severe metastatic lesions, and the magnitude of the disease is often influenced by factors that are independent from the parasite burden. Here, using *L*. *major* infected mice, we discovered that microbiota-dependent IL-17A–secreting ILCs promote increased disease early after infection. While the microbiota driven IL-17A–secreting ILCs mediated inflammatory response was independent of the parasite burden and a type 1 immune responses, it required stimulation of *Batf3*-dependent skin dendritic cells, production of IL-23 and the presence of neutrophils. This study provides mechanistic insight into how microbiota and the innate immune system influence pathology early after infection with *L*. *major*.

## Introduction

Cutaneous leishmaniasis includes a spectrum of diseases ranging from a single ulcerative lesion to severe metastatic lesions [1]. While control of these intracellular parasites is dependent upon the production of IFN-γ by CD4^+^ T cells, the magnitude of the disease is often influenced by factors other than the parasite burden. For example, even though *L*. *braziliensis* patients with classical cutaneous leishmanaisis mostly control the parasites, they develop chronic lesions that can be difficult to treat [2,3]. Several studies indicate that the magnitude of disease is often due to an uncontrolled inflammatory response, which can be mediated by IL-17A and/or IL-1β [1–4,5–10]. Using a combination of murine models and human studies we and others have shown that the skin microbiome enhances IL-1β and IL-17A production and contributes to increased pathology in cutaneous leishmaniasis [9,11,12]. However, while the role of T cells in promoting an increased inflammatory response is well established [1], whether innate cells initiate and/or amplify a pathogenic response in leishmaniasis is unknown.

Innate lymphoid cells (ILCs) comprise a family of lymphocytes, including ILC1s, ILC2s and ILC3s, that are quickly activated by multiple soluble signals for a rapid response to infection [13]. ILC1s produce IFN-γ in response against pathogens, while ILC2s produce IL-5 and IL-13 in response to allergic reactions. ILC3s mainly produce IL-17 and IL-22 and have important roles in epithelial tissue repair and inflammation [13]. ILCs have been extensively studied in the gut, and recently they have been shown to play a role in the pathology in the skin [14]. For example, ILC2s contribute to atopic dermatitis, while ILC3s are present in psoriatic lesions [14–17]. Although ILC3s secrete many of the same cytokines as Th17 cells, ILC3s have distinct functional and phenotypic features and respond in an antigen independent manner [13]. Importantly, microbial products and the local cytokine environment, such as IL-23 and IL-1α and/or IL-1β produced by myeloid cells, rapidly activate ILC3s to secrete IL-17 following bacterial exposure [13,18,19]. Thus, it seemed likely that ILCs might contribute to the pathologic host-microbiota interactions in cutaneous leishmaniasis.

Recent studies suggest that alterations to the skin microbiota, particularly changes in the dominance of *Staphylococcus* and *Streptococcus* species, at the site of *Leishmania* infection are linked to disease outcome [9]. Infection-induced alterations in the skin microbiota of *L*. *major* infected mice are linked to disease severity and immune-mediated inflammatory responses [9,20]. Additionally, *L*. *major* infection of germ-free (GF) mice results in smaller lesions and reduced immunopathology relative to specific pathogen free (SPF) mice [12]. Subsequent studies found that colonization with *Staphylococcus epidermidis* promoted the generation of IL-17 producing T cells [21], which in cutaneous leishmaniasis are associated with increased disease. However, the potential role of ILCs as a source of IL-17 in cutaneous leishmaniasis has not been explored.

To determine the role of ILCs in promoting pathology we investigated if *L*. *major* infection increased the number of IL-17-producing ILCs in the skin following infection. While infection did not induce early IL-17 production from T cells, we found an increase in IL-17 producing RORγt^+^ ILCs following infection. Furthermore, we found that topical colonization with murine or human skin isolates of *Staphylococcus* is associated with an increase in IL-17 producing ILCs and exacerbates immunopathology in *L*. *major* infected mice without altering type 1 immune responses. A critical role for IL-17 producing ILCs is suggested by our finding that these ILCs are able to promote increased pathology in the absence of T cells. Taken together, these studies indicate that the skin microbiota promotes accumulation of ILC3s that exacerbate IL-17-driven immunopathology in cutaneous leishmaniasis.

## Results

### IL-17-producing RORγt^+^ILCs are enriched in skin after *L*. *major* infection

**Fig 1:**
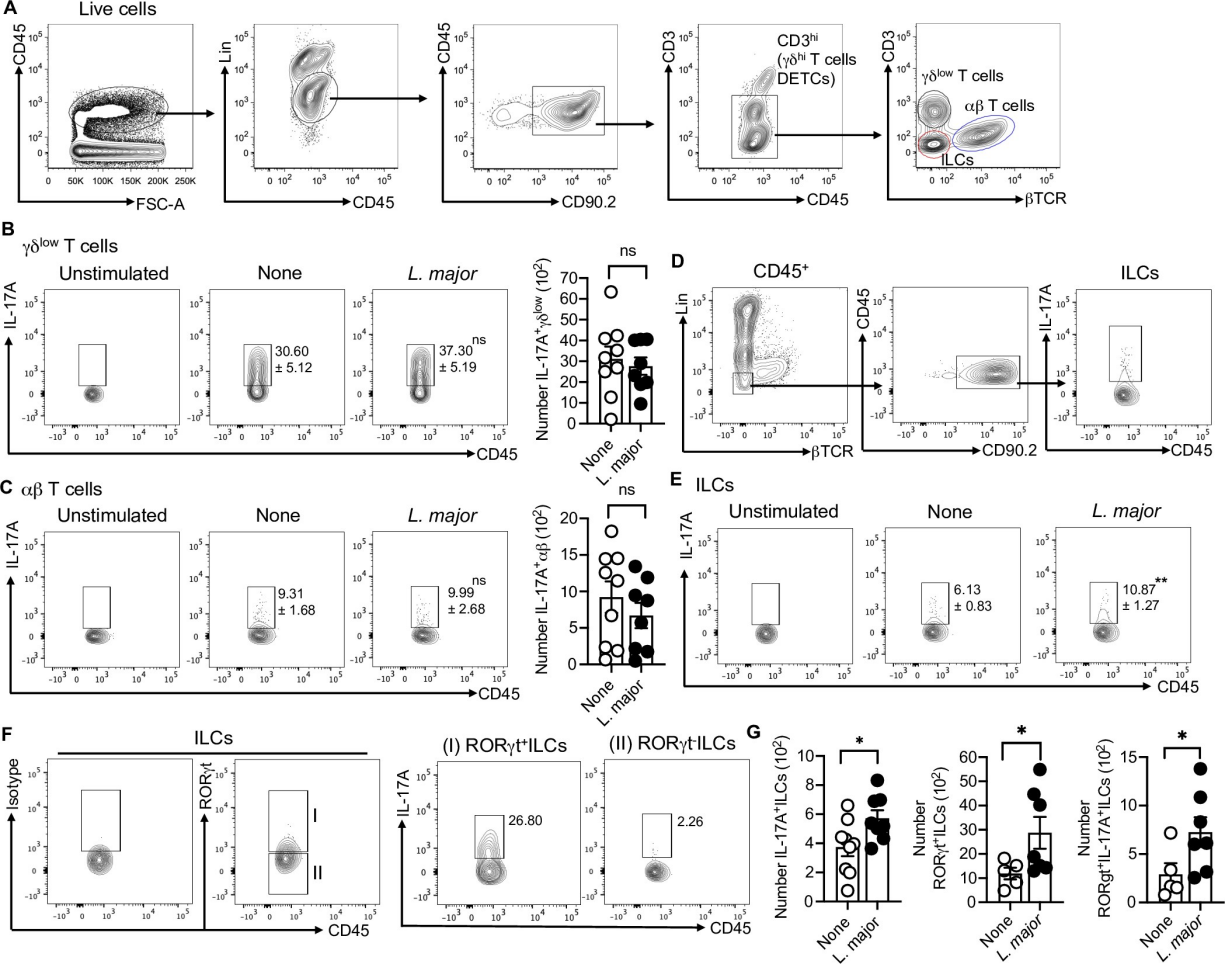
*L*. *major* infected skin contains IL-17A-producing RORγt^+^ILCs. **(A)** Gating strategy to identify skin T cells and ILCs. **(B)** Percent and number of IL-17Α^+^γδ^low^ T cells in the skin of uninfected (None) and *L*. *major* infected mice at week one. **(C)** Percent and number of IL-17Α^+^αβ T cells in the skin of uninfected (None) and *L*. *major* infected mice at week one. **(D)** Together with A, gating strategy to identify IL-17A^+^ILCs in skin. **(E)** Percent of IL-17Α^+^ILCs in the skin of uninfected (None) and *L*. *major* infected mice at week one. **(F)** Gating strategy to identify RORγt^+^IL-17A^+^ILCs in skin at week one. **(G)** Number of IL-17Α^+^ILCs, RORγt^+^ILCs and RORγt^+^IL-17A^+^ILCs in skin of uninfected (None) and *L*. *major* infected mice at week one. For intracellular staining cells were stimulated with PMA/Ion for 4 hours. Unstimulated cells were used as control to define the gates (B,C,E). Data are from two experiments with a total of five to nine mice in each group (B,C,E,G). Number within the flow plot show percent of IL-17A^+^ cells with SEM (B,C,E). Error bars shows SEM. Two-tailed unpaired Student’s t-test with Welch’s correction. *p<0.05, **p<0.01, ***p<0.001.

IL-17 has been shown to play an important role in mediating inflammation in cutaneous leishmaniasis [7,8,22] and while Th17 cells are a source of IL-17, it is also possible that IL-17 produced by ILCs present in skin could contribute to immunopathology. To assess the potential role of IL-17 from ILCs early on in *L*. *major* infected skin, we analyze IL-17 from skin ILCs and T cells one-week post infection **(Fig 1A)**. In our analysis of the cytokine IL-17 following *L*. *major* infection, we found no differences in IL-17 production from skin γδ^low^ T cells and αβ T cells compare to control mice at week one post infection **(Fig 1B and 1C)**. However, the ILC3 signature cytokine IL-17 was increased in ILCs of *L*. *major* infected mice compared to non-infected mice. RORγt is expressed by ILC3s that produce IL-17 [14]. To determine whether these IL-17-producing ILCs in *L*. *major* infected skin were RORγt^+^, we examined IL-17 production from RORγt^+^ ILCs and RORγt^-^ ILCs. We found that almost all IL-17 produced by RORγt^+^ ILCs, and the number of RORγt^+^ ILCs, was significantly higher in *L*. *major* infected compared to control mice **(Fig 1D–1G)**. Consistently, we also found a significant increase in the number of RORγt^+^ IL-17A^+^ ILCs in *L*. *major* infected compare to control mice one-week post infection **(Fig 1G)**. Moreover, we did not see induction of IFN-γ and IL-13 production from ILCs **(S1A–S1D Fig)**. We also did not see any induction in IFN-γ production from γδ^low^ T cells and αβ T cells in the lesions following *L*. *major* infection compared to uninfected mice at week one **(S2A–S2D Fig)**. Together, these data suggest that RORγt^+^ILCs are an early source of IL-17 in the skin following acute infection by *L*. *major* and may contribute to the immunopathology of cutaneous leishmaniasis.

### *S*. *epidermidis* enhances skin inflammation and IL-17-producing RORγt^+^ILCs in *L*. *major* infection

**Fig 2:**
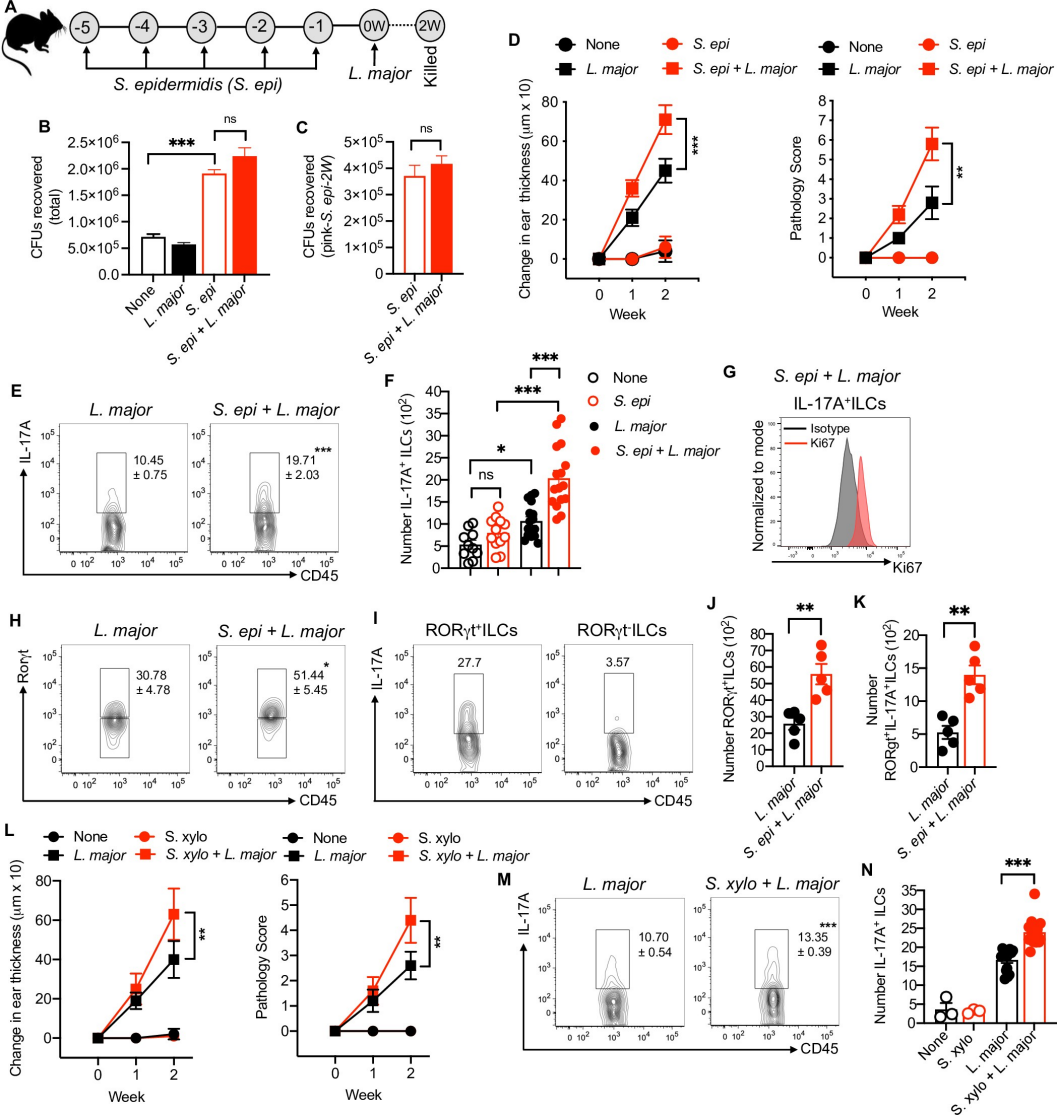
. *S*. *epidermidis and S*. *xylosus* colonization before *L*. *major* infection increase the inflammatory responses and IL-17A^+^ILCs. **(A)** Schematic representation of *S*. *epidermidis* ***(****S*. *epi*) and *L*. *major* treatment protocol in C57BL/6 mice. **(B)** Recovered total colony forming units (CFUs) in the ear of different treatment groups at week two. **(C)** Recovered pink (*S*. *epi*-2W) colony forming units (CFUs) in the ear of different treatment groups at week two. **(D)** Ear thickness measurement and pathology score in mice associated with *S*. *epi* or unassociated prior to *L*. *major* infection at week one and two. **(E)** Percent of IL-17Α^+^ILCs in the skin of different treatment groups at week two. **(F)** Number of IL-17Α^+^ILCs in the skin of different treatment groups at week two. **(G)** Ki67 staining on IL-17A^+^ ILCs in *S*. *epi* colonized and *L*. *major* infected mice at week two. Total ILCs were used as isotype. **(H)** Percent of RORγt^+^ILCs in the skin of different treatment groups at week two. **(I)** IL-17A production from RORγt^+^ILCs and RORγt^-^ILCs in *S*. *epi* colonized and *L*. *major* infected mice at week two. **(J)** Number of RORγt^+^ILCs in the skin of different treatment groups at week two. **(K)** Number of RORγt^+^IL-17A^+^ILCs in the skin of different treatment groups at week two. **(L)** Ear thickness measurement and pathology score in mice associated with *S*. *xylosus (S*. *xylo)* or unassociated prior to *L*. *major* infection at week one and two. **(M, N)** Percent and number of IL-17Α^+^ILCs in the skin of different treatment groups at week two. The number within the flow plots show percent of IL-17A^+^ cells (E,M) or RORγt^+^ cells (H) with SEM or IL-17A^+^ cells with in the gated box (I). Data are from three experiments with a total of 10 to 16 mice in each group (E,F,M,N) except control groups in N which is from one experiment with three mice or from one experiment representative of two with five mice in each group (B,C,D,L) or from one experiment with three to five mice in each group (G,H,J,K). Error bars shows SEM (B,H,I.J,N) or SD (D,L). Two-tailed unpaired Student’s t-test with Welch’s correction or one-way ANOVA with Tukey’s multiple comparison analysis (B,F,N). ns, not significant; *p<0.05, **p<0.01, ***p<0.001.

The skin microbiota contribute to the severity of human inflammatory skin diseases and our previous findings revealed a link between *Staphylococcus* and more severe disease in *L*. *major* infected mice [9]. To determine if skin colonization with *Staphylococcus* influences immunopathology by impacting IL-17-producing ILCs, we topically applied *S*. *epidermidis* to the ears and back skin of the mice once daily for five days (**Fig 2A**). We quantified skin colonization by counting total CFUs and *Staphylococcus epidermidis* (based on the expression of mCherry) CFUs at week two (**Fig 2B and 2C**). One day after the last treatment with *S*. *epidermidis*, mice were infected in the ear with *L*. *major* and monitored for six weeks **(S3A Fig)**. We found that *S*. *epidermidis* colonization before *L*. *major* infection significantly increased the inflammatory response as compared to *L*. *major* infected mice alone (**Figs 2D and S3B**). In contrast, *S*. *epidermidis* association without *L*. *major* infection did not elicit an inflammatory response **(Figs 2D and S3B)**. Flow cytometry analysis revealed that *S*. *epidermidis* colonization before *L*. *major* infection increased the early IL-17^+^ ILCs around two-fold compared to *L*. *major* alone at week two (**Fig 2E and 2F**). The increase in the number of IL-17A^+^ ILCs in *L*. *major* infected and colonized mice may be due to the proliferation of this subset in the skin as indicated by Ki67 staining of IL-17A^+^ILCs **(Fig 2G)**. Moreover, RORγt^+^ ILCs were significantly increased in *S*. *epidermidis* colonized and *L*. *major* infected mice compared to *L*. *major* infected mice alone. Consistently, in analyzing IL-17 production from RORγt^+^ ILCs and RORγt^-^ ILCs, we found that almost all IL-17 was produced by RORγt^+^ ILCs in *S*. *epidermidis* colonized and *L*. *major* infected mice and the number of RORγt^+^ IL-17A^+^ ILCs was increased in these mice compare to *L*. *major* alone **(Fig 2H–2K)**. In contrast, we did not see substantial accumulation of IL-17A^+^ ILCs in infected groups at the peak of infection (5 weeks) with only a modest increase in *S*. *epidermidis* colonized and *L*. *major* infected mice compare to *L*. *major* alone in the skin **(S3C and S3D Fig)**. These results suggest that IL-17A^+^ ILCs may be most important during the early phase of the infection. Next, we extended our findings using an isolate of *Staphylococcus xylosus* that was obtained from *L*. *major* infected mice that is commonly found on uninfected murine skin and wounds [23,24]. Consistently, *S*. *xylosus* colonization before *L*. *major* infection led to an increased inflammatory response as determined by skin thickness and pathology score compared to *L*. *major* infected mice without colonization **(Fig 2L)**. In addition, *S*. *xylosus* colonization also increased the number of IL-17A^+^ ILCs compared to uncolonized *L*. *major* infected mice at week two **(Fig 2M and 2N).** These data suggest that murine and human skin commensals induce RORγt^+^ IL-17A^+^ ILCs that contribute to the inflammatory responses in the skin during early cutaneous leishmaniasis.

### *S*. *epidermidis* does not impact type 1 immune responses

**Fig 3:**
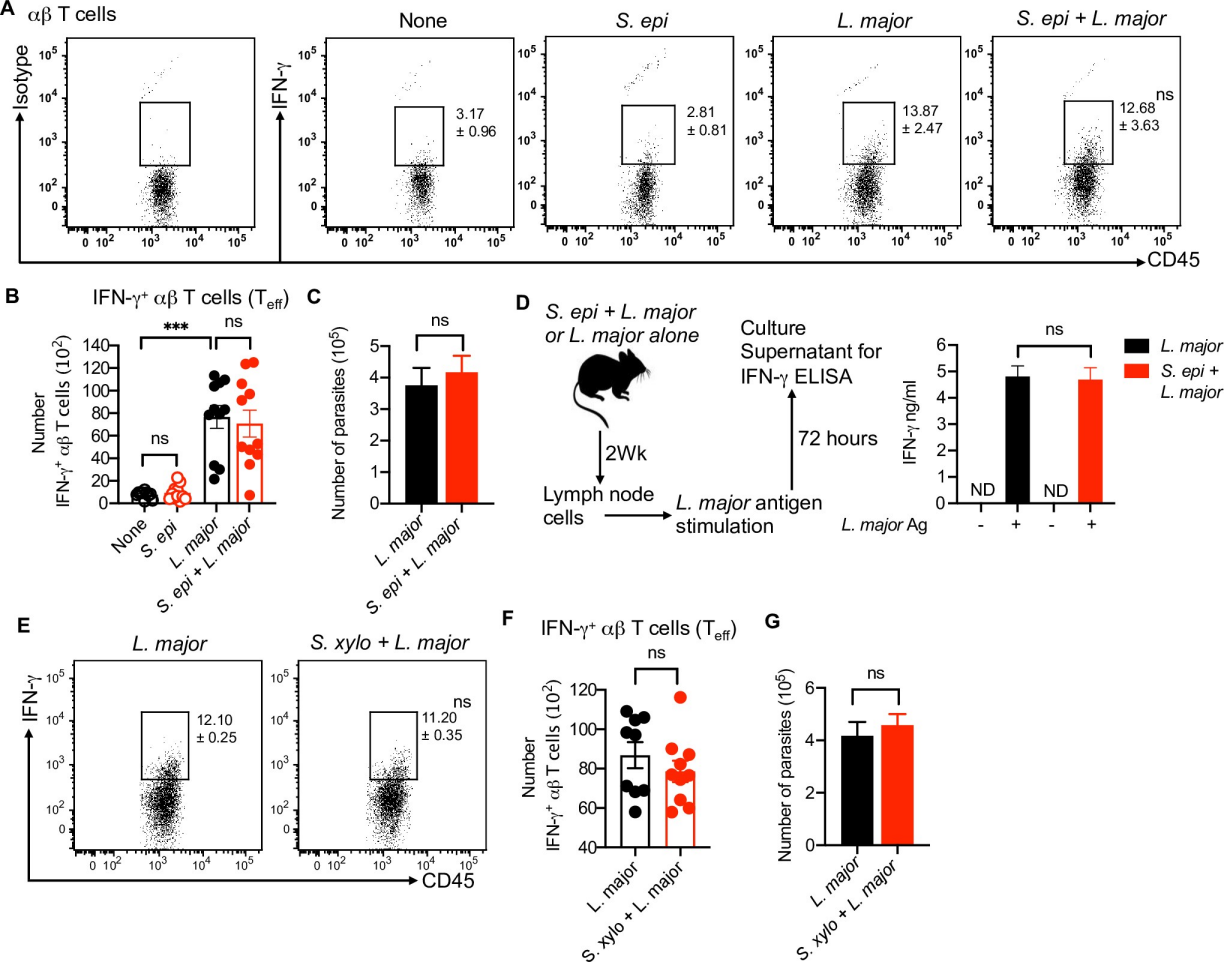
*S*. *epidermidis and S*. *xylosus* colonization does not impact the type 1 immune response. **(A)** Percent of IFN-γ- producing αβ T cells in the skin of different treatment groups at week two. **(B)** Number of IFN-γ- producing αβ T cells in the skin of different treatment groups at week two. **(C)** *L*. *major* parasite load in different treatment groups at week two. **(D)** *L*. *major* antigen specific IFN-γ level in cultured supernatant of lymph node cells in different treatment groups. Cells were prepared from mice at week two. **(E)** Percent of IFN-γ-producing αβ T cells in the skin of different treatment groups at week two. **(F)** Number of IFN-γ- producing αβ T cells in the skin of different treatment groups at week two. **(G)** *L*. *major* parasite load in different treatment groups at week two. Number within the flow plot show percent of IFN-γ^+^ cells with SEM (A,E). Data are from three (A,B) or two (C,D,E,F,G) experiments with a total of five to 12 mice in each group. Statistical comparison within the flow plot is with the *L*. *major* group. Error bars shows SEM. Two-tailed unpaired Student’s t-test with Welch’s correction or one-way ANOVA with Tukey’s multiple comparison analysis (B). ND, not detected; ns, not significant; ***p<0.001.

To assess if commensal bacterial colonization of the skin alters type 1 immunity in our studies, we colonized the mice with *S*. *epidermidis* for five days as described above and then infected the mice with *L*. *major*. At two weeks post-infection lesions were analyzed for immune responses and parasite burden. There was no difference in skin IFN-γ production from αβ T cells in *S*. *epidermidis* colonized and *L*. *major* infected mice compare to *L*. *major* infected mice alone (**Fig 3A and 3B**), and the numbers of *L*. *major* parasites were similar in both groups **(Fig 3C)**. We also assessed *L*. *major* specific IFN-γ by activating the cells from skin draining lymph node with *L*. *major* antigen *in vitro* and found no difference in IFN-γ production in *S*. *epidermidis* colonized and *L*. *major* infected mice compared to *L*. *major* infected mice **(Fig 3D)**. Next, we similarly evaluated the effect of *S*. *xylosus* on skin IFN-γ production from αβ T and parasite load; mice colonized with *S*. *xylosus* before *L*. *major* infection exhibited no difference in IFN-γ production from αβ T cells or any difference in parasite burden **(Fig 3E–3G)**. Overall, these results suggest that the skin commensals that we used in this study do not alter type 1 immune responses during the early phase of the infection.

### *S*. *epidermidis* dependent IL-17-producing ILCs and inflammation require CD103^+^ dendritic cells

**Fig 4:**
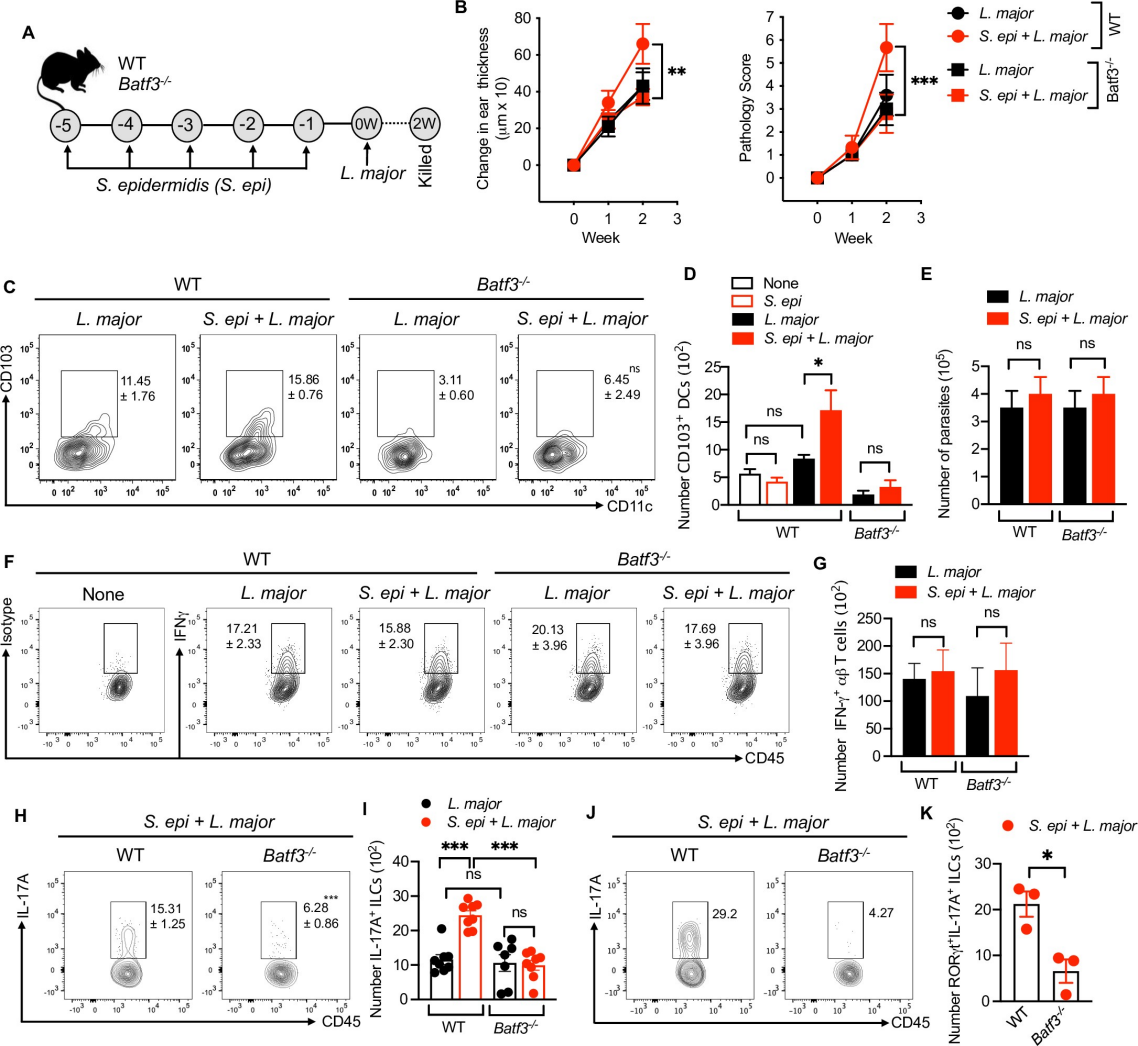
*S*. *epidermidis* dependent IL-17A^+^ILCs and inflammatory responses require CD103^+^ DCs. **(A)** Schematic representation of *S*. *epi* and *L*. *major* treatment protocol in WT and *Batf3*^*-/-*^ mice. **(B)** Ear thickness measurement and pathology score in WT and *Batf3*^*-*/-^ mice colonized with *S*. *epi* or uncolonized prior to *L*. *major* infection at week one and two. **(C,D)** Percent and number of CD103^+^ DCs at week two in WT and *Batf3*^*-*/-^ mice colonized with *S*. *epi* or uncolonized prior to *L*. *major* infection at week two. **(E)** *L*. *major* parasite load in WT and *Batf3*^*-*/-^ mice colonized with *S*. *epi* or uncolonized prior to *L*. *major* infection at week two. **(F,G)** Percent and number of IFN-γ^+^αβ T cells in WT and *Batf3*^*-*/-^ mice colonized with *S*. *epi* or uncolonized prior to *L*. *major* infection at week two. **(H,I)** Percent and number of IL-17A^+^ILCs in WT and *Batf3*^*-*/-^ mice colonized with *S*. *epi* or uncolonized prior to *L*. *major* infection at week two. **(J,K)** Percent and number of RORγt^+^IL-17A^+^ILCs in the skin of *S*. *epi* colonized and *L*. *major* infected WT and *Batf3*^*-/-*^ mice at week two. Number within the flow plot show percent of CD103^+^CD11c^+^ (C), IFN-γ^+^ (F) and IL-17A^+^ (H,J) cells with SEM. Data are from two experiments with a total of six to eight mice in each group (C,D,F,G,H,I) or from one experiment representative of two with five mice in each group (B,E). Statistical comparison with in the flow plot in C is with *L*. *major* group of *Batf3*^*-/-*^ mice. Error bars shows SEM (D,E,G,I,J,K) or SD (B). Two-tailed unpaired Student’s t-test with Welch’s correction or one-way ANOVA with Tukey’s multiple comparison analysis (D,I). ns, not significant; *p<0.05, **p<0.01, ***p<0.001.

Microbes and their products are potent inducers of innate and adaptive immune cell responses driven by dendritic cells (DCs). The skin contains different subsets of DCs that drive unique immune responses, and previous reports indicate that CD103^+^ DCs in skin are the primary sensor of commensals that regulate formation of commensal-specific T-cell responses [21]. CD103^+^ DCs are classical CD11c^+^CD11b^-^ DCs that depend on the transcription factor *Batf3* for their development in the skin [21]. Therefore, we employed *Batf3*^*-/-*^ mice to explore whether or not CD103^+^ DCs are required for IL-17-producing ILC responses and enhanced immunopathology due to *S*. *epidermidis* colonization during *L*. *major* infection (**Fig 4A**). The lack of CD103^+^ DCs did not affect the lesion size in the *Batf3*^*-/-*^ mice compared to wild-type mice infected with *L*. *major* alone. However, the absence of CD103^+^ DCs completely abrogated the *S*. *epidermidis* mediated effect on immunopathology during *L*. *major* infection, as assessed by ear thickness and pathology score (**Fig 4B)**. As expected, *L*. *major* infected *Batf3*^*-/-*^ mice, with or without colonization, mostly lack CD103^+^ DCs in the skin (**Fig 4C and 4D**). In contrast, topical association with *S*. *epidermidis* increased the number of CD103^+^ DCs in skin of WT mice after *L*. *major* infection (**Fig 4D**).

To determine if the CD103^+^ DC-dependent effects of *S*. *epidermidis* colonization on *L*. *major* induced pathology were due to the lack of a type 1 immune response, we next evaluated the parasite burden and the number of adaptive immune cells in the lesion. CD103^+^ DCs have been shown to be critical for the production of IL-12 in leishmaniasis, and *Batf3*^*-/-*^ mice develop an uncontrolled infection over time [25]. However, similar to what has been previously reported [26], at this early time point parasite number and the number and percent of IFNγ^+^ αβ T cells in the skin of WT and *Batf3*^*-/-*^ mice were similar **(Fig 4E–4G)** indicating that the lack of CD103^+^ DC did not impact early type 1 immune responses or control of *L*. *major* parasites. Furthermore, we analyzed IL-17-producing ILCs in WT and *Batf3*^*-/-*^ mice infected with *L*. *major* alone or in conjunction with *S*. *epidermidis* colonization. While there was no difference in the number of IL-17^+^ ILCs in WT and *Batf3*^*-/-*^ mice infected with *L*. *major* alone, the number of IL-17^+^ ILCs was significantly reduced in *L*. *major* infected *Batf3*^*-/-*^ mice colonized with *S*. *epidermidis* when compared to similarly treated WT mice **(Fig 4H and 4I)**. Similarly, the number of RORγt^+^IL-17A^+^ ILCs was also decreased in *L*. *major* infected *Batf3*^*-/-*^ mice colonized with *S*. *epidermidis* compared to WT mice (**(Fig 4J and 4K**). In addition, the total number of ILCs and Ki67^+^ ILCs were increased in *S*. *epidermidis* colonized and *L*. *major* infected mice compare to control (None) and the number was not changed between the WT and *Batf3*^*-/-*^ mice in treatment groups suggesting that the decrease in IL-17A^+^ ILCs in *Batf3*^*-/-*^ mice is not due to an overall decrease in ILCs **(S4A and S4B Fig)**. These data suggest that *S*. *epidermidis* mediated IL-17-producing ILC responses are dependent on skin CD103^+^ DCs and support the idea that microbes stimulate CD103^+^ DCs to drive the induction and/or maintenance of IL-17-producing ILCs that contribute to early immunopathology in cutaneous leishmaniasis.

### *S*. *epidermidis* dependent IL-17-producing ILCs require IL-23

**Fig 5:**
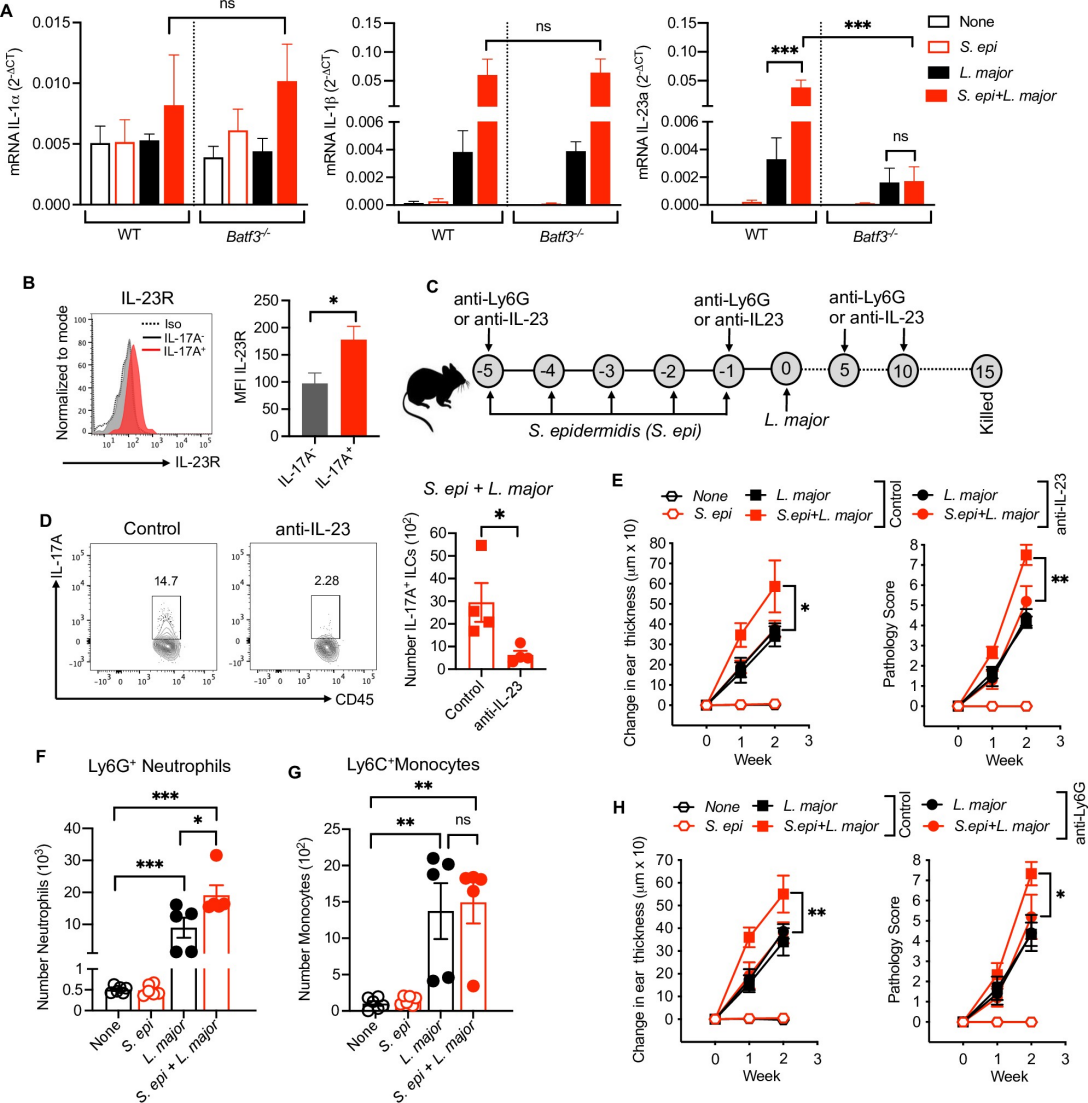
*S*. *epidermidis* dependent IL-17A^+^ILCs require IL-23. **(A)** qRT-PCR analysis of IL-1α, IL-1β and IL-23a in WT and *Batf3*^*-/-*^ mice in different treatment groups at week two. **(B)** IL-23R expression on IL-17A^+^ and IL-17A^-^ILCs in *S*. *epi* colonized and *L*. *major* infected mice at week two. **(C)** Schematic representation of anti-IL-23 and anti-Ly6G treatment protocol in *S*. *epi* colonized and *L*. *major* infected WT mice. **(D)** IL-17A production from ILCs in control (IgG) and anti-IL-23 treated in *S*. *epi* colonized and *L*. *major* infected at week two. **(E)** Ear thickness measurement and pathology score in IgG and anti-IL-23 treated *L*. *major* and in *S*. *epi* colonized and *L*. *major* infected mice at week one and two. **(F,G)** Number of neutrophils and monocytes in different treatment groups at week two. **(H)** Ear thickness measurement and pathology score in IgG and anti-Ly6G treated *L*. *major* and in *S*. *epi* colonized and *L*. *major* infected mice at week one and two. Number within the flow plot show percent of IL-17A^+^ cells within the gated box. Data are from one experiment with a total of three to five mice in each group (A,B,D,E,F,G,H) Error bars show SEM (A,D,F) or SD (E,H). Two-tailed unpaired Student’s t-test with Welch’s correction or one-way ANOVA with Tukey’s multiple comparison analysis (A,F,G). ns, not significant; *p<0.05, **p<0.01, ***p<0.001.

Previous reports suggest that IL-17 responses in ILCs is mediated by IL-23 [14]. Το determine if IL-23 was responsible for the induction of IL-17 from the ILCs after *S*. *epidermidis* colonization in *L*. *major* infected mice, we performed mRNA analysis of IL-1α, IL-1β, IL-12, IL-23 and IL-33 in lesions between the treatment groups **(Figs 5A and S5)**. The mRNA of both IL-1β and IL-23 was increased in colonized and *L*. *major* infected mice compared to *L*. *major*. Additionally, in *L*. *major* infected mice IL-1β and IL-23 gene expression was modestly increased compared to *S*. *epidermidis* or non-treated groups **(Figs 5A and S5)**. In analyzing these mRNA transcripts in *Batf3*^*-/-*^ mice, we found that IL-23 but not IL-1β was significantly reduced in *Batf3*^*-/-*^ mice compared to WT mice infected with *S*. *epidermidis* and *L*. *major*. However, we did not see any significant change in IL-1α, IL-12 and IL-33 between the groups **(Figs 5A and S5)**. Furthermore, we performed flow analysis of IL-1R, IL-12R, IL-23R and IL-33R on IL-17A^+^ and IL-17A^-^ ILCs in *S*. *epidermidis* colonized and *L*. *major* infected mice. We detected higher level of IL-23R and IL-1R expression on IL-17A^+^ ILCs compared to IL-17A^-^ ILCs. IL-33R was expressed on IL-17A^-^ ILCs and IL-12R was not detected on either subset of ILCs **(Figs 5B and S6)**. To determine if IL-23 was required for the induction of IL-17A in ILCs and inflammatory responses, we treated mice with anti-IL-23 mAb **(Fig 5C)**. Neutralization of IL-23 decreased IL-17A^+^ ILCs, lesion size and pathology in mice colonized with *S*. *epidermidis* and infected with *L*. *major*. In contrast, there was no effect in *L*. *major* infected mice alone suggesting that inflammatory responses caused by *L*. *major* infection are independent of IL-23 **(Fig 5D and 5E)**. Moreover, to determine if neutrophil recruitment in response to IL-17 is responsible for the pathology, we quantified Ly6G^+^ neutrophils together with Ly6C^+^ monocytes between the treated groups. The number of neutrophils was significantly increased in *L*. *major* infected mice compared to controls which was further increased in colonized and infected mice. In contrast, the number of monocytes was not increased in *S*. *epidermidis* colonized and *L*. *major* infected mice compare to *L*. *major* alone **(Fig 5F and 5G)**. Furthermore, neutrophil depletion did not affect the pathology in *L*. *major* infected mice, but significantly reduced the pathology in mice colonized with *S*. *epidermidis* and infected with *L*. *major* **(Fig 5H)**. These data suggest that *S*. *epidermidis* stimulate *Batf3*-dependent IL-23 production which is required for the IL-17 production from ILCs in *L*. *major* infected skin to mediate neutrophil dependent pathology.

### IL-17^+^ ILCs are sufficient in mediating early *S*. *epidermidis* dependent inflammation

**Fig 6:**
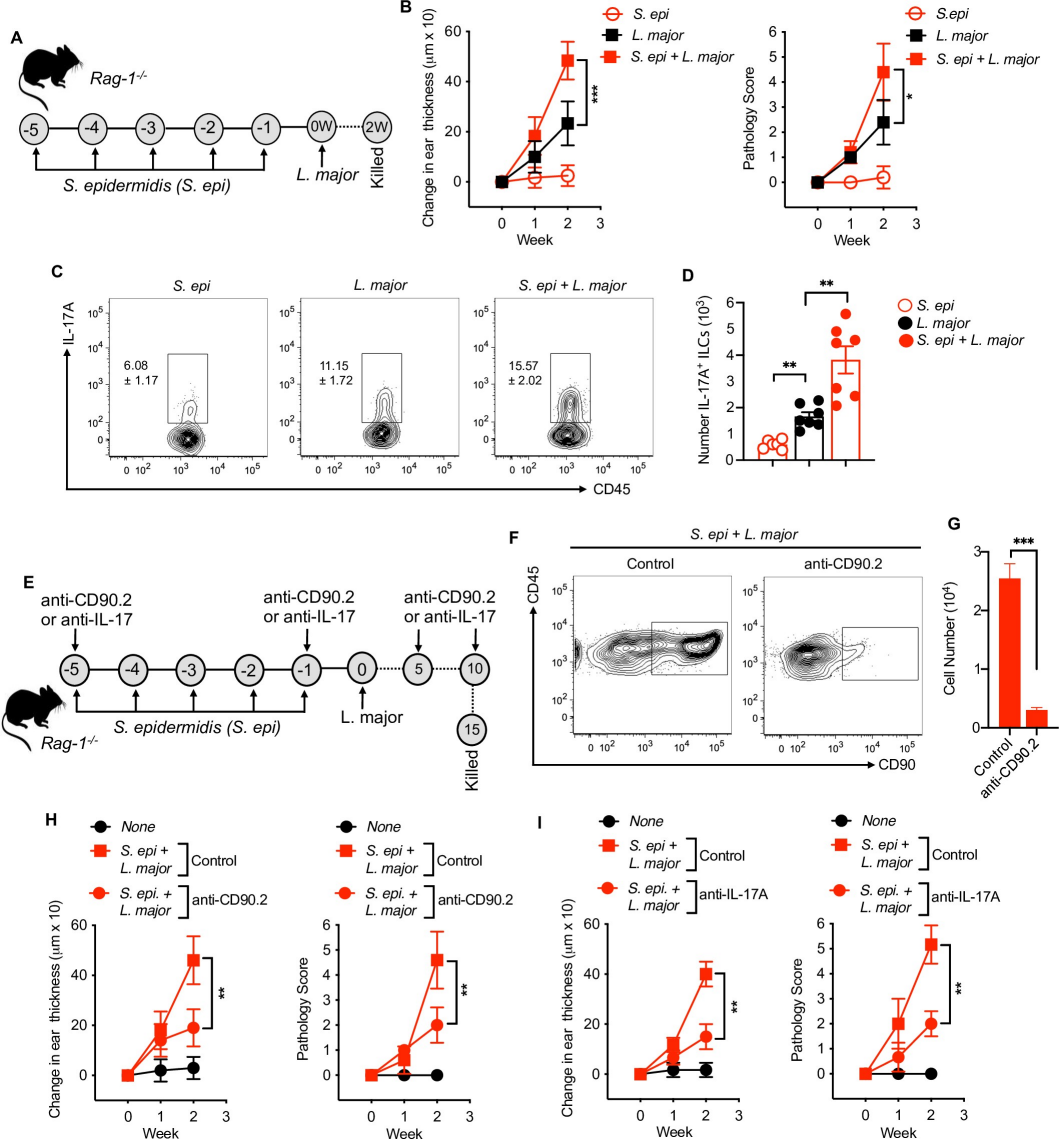
IL-17A^+^ILCs are sufficient to drive *S*. *epidermidis* mediated inflammation. **(A)** Schematic representation of *S*. *epi* and *L*. *major* treatment protocol in *Rag1*^*-/-*^ mice. **(B)** Ear thickness measurement and pathology score in *Rag1*^*-/-*^ mice colonized with *S*. *epi* or uncolonized prior to *L*. *major* infection at week one and two. **(C,D)** Percent and number of IL-17A- producing ILCs at week two in *Rag1*^*-/-*^ mice colonized with *S*. *epi* or uncolonized prior to *L*. *major* infection. **(E)** Schematic representation of ILC depletion or IL-17 neutralization treatment protocol in *Rag1*^*-/-*^ mice. **(F)** Representative flow cytometry plot of ILC depletion as identified as CD45^+^CD90^+^ cells in *Rag1*^*-/-*^ mice. **(G)** Number of CD90^+^ cells in control and anti-CD90.2 treated mice. **(H)** Ear thickness measurement and pathology score in *Rag1*^*-/-*^ mice with and without (Control; IgG) ILC depletion in different groups. **(I)** Ear thickness measurement and pathology score in *Rag1*^*-/-*^ mice with and without (Control; IgG) IL-17A neutralization by anti-IL-17A antibody in different groups. Number within the flow plot show percent of IL-17A^+^ cells with SEM (C). Data are from two experiments with a total of six to seven mice in each group. Statistical comparison within the flow plot is with *L*. *major* group. Error bars shows SEM (D,G) or SD (B,H,I). Two-tailed unpaired Student’s t-test with Welch’s correction. *p<0.05, **p<0.01, ***p<0.001.

To determine if IL-17 from ILCs can drive the inflammatory responses in the absence of T cells, we colonized *Rag1*^*-/-*^ mice with *S*. *epidermidis* and then infected them with *L*. *major* or infected uncolonized mice with *L*. *major* **(Fig 6A)**. Colonization with *S*. *epidermidis* increased the ear thickness and pathology in *Rag1*^*-/-*^ mice compared to *L*. *major* infected mice alone **(Fig 6B)**. In analyzing the IL-17^+^ILCs in different treatment groups, we found that *S*. *epidermidis* colonization significantly increased the IL-17^+^ILCs in *L*. *major* infected *Rag1*^*-/-*^ mice compared to uncolonized mice **(Fig 6C and 6D).** Of note, analysis of IFN-γ and IL-5 production in treatment groups revealed no difference in IFN-γ and reduced IL-5 from ILCs in *S*. *epidermidis* colonized and *L*. *major* infected mice compared to uncolonized mice **(S7A and S7B Fig)**.

To directly demonstrate that ILCs were promoting the increased immunopathology following *L*. *major* infection in *Rag1*^*-/-*^ mice, we depleted ILCs by injecting anti-CD90.2 antibody into *Rag1*^*-/-*^ mice just before the first *S*. *epidermidis* colonization and then at days 5 and 10 after *L*. *major* infection **(Fig 6E)**. Depletion of CD90.2 cells in *Rag1*^*-/-*^ mice was confirmed by flow cytometry **(Fig 6F and 6G)**. Depleting CD90.2^+^ ILCs significantly reduced the inflammatory responses as assessed by ear thickness and pathology score two weeks post-infection in *S*. *epidermidis* colonized and *L*. *major* infected *Rag1*^*-/-*^ mice compared to *S*. *epidermidis* colonized and *L*. *major* infected *Rag1*^*-/-*^ mice that did not receive the anti-CD90.2 antibody (‘control’) **(Fig 6H**) To test that IL-17 production by ILCs was responsible for mediating the pathology in *Rag1*^*-/-*^ mice, we treated mice with anti-IL-17A mAb. Notably, blockade of IL-17A in *Rag1*^*-/-*^ mice colonized with *S*. *epidermidis* before *L*. *major* infection significantly reduced the inflammation (**Fig 6I)** suggesting that IL-17 production from ILCs is sufficient to drive inflammation in acute phase of cutaneous leishmaniasis. In comparing the inflammatory responses in *L*. *major* and *S*. *epidermidis* colonized and *L*. *major* infected WT and *Rag1*^*-/-*^ mice, we observed lower inflammatory responses in *Rag1*^*-/-*^ mice compared to WT in both treatment groups. However the relative increase was the same between the groups, suggesting that the augmented pathology in colonized and infected mice was largely IL-17A^+^ ILC dependent **(S7A–S7C Fig)**. Together, these results further support that IL-17-producing RORgt^+^ ILCs are critical mediators of microbiota-driven inflammatory responses early after infection in cutaneous leishmaniasis.

## Discussion

Cutaneous leishmaniasis exhibits a wide spectrum of clinical presentations, and understanding the mechanisms driving these diverse manifestations is critical for the development of new therapies. In some cases, the lack of an effective immune response leads to uncontrolled parasite replication leading to severe disease [1]. However, in other situations effective immunity develops, but an exaggerated inflammatory response sustains disease [1]. Here we report one pathway leading to increased disease without an increase in the parasite burden that is mediated by bacterial colonization and the subsequent expansion of a pathologic IL-17-producing RORγt^+^ILC population in the skin.

It appears that several pathways can lead to pathologic inflammatory responses in cutaneous leishmaniasis, including extensive cell lysis leading to inflammasome activation and IL-1β production [6,27–29], infection with more virulent strains of the parasite [5] and the lack of regulatory cytokines such as IL-10 leading to an IL-1β and IL-17 mediated pathology [7,30]. Additionally, alterations in the skin microbiome influence disease. We found that during *L*. *major* infection of mice there is a dramatic decrease in bacterial diversity in the skin that coincides with increases in *Staphylococcus* relative abundance. In co-housing experiments we demonstrated that transfer of this lesion-associated microbiota promotes increased severity of leishmanial lesions [9]. Correspondingly, germ-free mice develop smaller lesions than conventional mice, and colonization of mice with *Staphylococcus* leads to increased disease [12]. Thus, while alterations to the microbiota can be a consequence of inflammation and tissue damage, these studies demonstrate the potential for the skin microbiota to promote inflammation. Alterations to the skin microbiome occur in both murine and human cutaneous leishmaniasis [9,31], and our studies indicate how those alterations impact IL-17-producing RORγt^+^ILCs and their contributions to immunopathology in cutaneous leishmaniasis.

ILCs are a heterogeneous group of innate immune cells: ILC1s secrete IFN-γ, ILC2s secrete IL-5 and IL-13, and ILC3s secrete IL-17 and IL-22 [32]. As the role of ILCs has not been investigated in leishmaniasis, we first asked whether the acute infection altered the ILCs in the skin. Since *L*. *major* infection in mice that resolve the disease is associated with a CD4 Th1 response, we predicted that there might be an increase in IFN-γ from ILCs early after infection. Consistent with this prediction was our previous finding that NK cells in *L*. *major* infected resistant mice contribute to an early Th1 response in some mouse strains [33]. Here we analyzed ILCs excluding NK1.1^+^ subset, and we found no significant changes or induction of IFN-γ or IL-13 production from ILCs after infection, suggesting that these cytokine secreting ILCs probably are not major players in the early immune response to *L*. *major* in C57BL/6 mice. Whether they might play a role in other resistant strains is unknown. In contrast, we observed, an increase in ILCs producing IL-17 that was driven by *L*. *major* infections and further enhanced by bacteria colonization of the skin, leading to increased disease. IL-17-producing ILCs (ILC3s) are present at barrier surfaces in close contact with the microbiota, which can have a profound effect on tissue inflammation and homeostasis [13,14]. Further, it is known that the gut and skin commensal microbiota play an important role in regulating IL-17 responses in ILC3 [14,19], which is consistent with our findings. Such results indicate that the endogenous skin microbiota will likely impact responses to both infection and vaccination and that therapeutic modulation of the commensal microbiota in skin could potentially be harnessed to increase vaccine efficacy [34,35].

IL-17A has been implicated in the immunopathology of several experimental models of cutaneous leishmaniasis. Notably, *S*. *aureus* infection together with *L*. *major* exacerbates the IL-17A dependent pathology [11], and cytoplasmic virus within a strain of *L*. *guyanensis* induces IL-17A production to mediate disease severity [36]. We extend the current understanding of immunopathology in cutaneous leishmaniasis by demonstrating that commensal microbiota induced IL-17A-producing RORγt^+^ ILCs drive the early pathology in *L*. *major* infected mice via recruitment of neutrophils to the site of infection. In support of a pathogenic role for these cells in the skin, IL-17A producing RORγt^+^ ILCs have been implicated in models of psoriasis [15,37,38]. Furthermore, the increased number of IL-17-producing ILC3s in lesions of psoriatic patients was positively correlated with disease severity, and adoptive transfer of ILC3s that can produce IL-17 in a human xenotransplant mouse model was sufficient to induce psoriasis [37]. By using *Rag1*^*-/-*^ mice which lack T and B cells, we directly demonstrated the importance of IL-17^+^ILCs and commensal microbiota in mediating the early skin pathology in *L*. *major* infection, as IL-17A neutralization or CD90.2 depletion in *Rag1*^*-/-*^ mice reduced the pathology in *L*. *major* infected mice associated with *S*. *epidermidis*. *Rag1*^*-/-*^ mice also lack the Tregs, and current studies are examining their potential role in controlling the IL-17A response. In addition, as was expected lesions from *L*. *major* and *L*. *major* colonized *Rag1*^*-/-*^ mice were smaller than WT mice, but even in the absence of T cells colonization influenced lesion development. However, T cells are critical to achieve full inflammatory responses/pathology in WT mice. Together, this study suggests that the role of IL-17-producing RORγt^+^ ILCs in inducing inflammatory responses is not limited to inflammatory skin conditions such as psoriasis but contributes to other diseases such as cutaneous leishmaniasis.

We found that *Staphylococcus* colonization increased lesion size and the degree of pathology in mice infected with *L*. *major*, while not influencing the parasite load. We observed this result both with *S*. *epidermidis* and a strain of *S*. *xylosus* that we isolated from mice infected with *L*. *major* [9]. These results are similar to our previous findings where co-housed mice had more severe disease, but no change in the parasite burden [9]. Since the parasite burden does not change, it is not surprising that there was no alteration in the IFN-γ response. However, it is clear that in some situations the microbiota can influence IFN-γ responses. In one study the total lack of microbiota in germ-free C57BL/6 mice infected with *L*. *major* led to low levels of IFN-γ, suggesting that some threshold of microbiota may be required for optimal IFN-γ responses [12]. However, contradictory results were obtained in another study, where the parasite burden was higher in germ-free mice, while the levels of IFN-γ were similar between SPF and germ-free mice infected with *L*. *major* [39]. These differences may be due to differences in the genetic background of the germ-free mice, the route of infection, or the strain or species of leishmania studied [12,20,39].

The pathway leading to expansion of IL-17^+^ RORγt^+^ ILCs following bacterial association and *L*. *major* infection may be similar to that driving T cell production of IL-17. We found that *Batf3* dependent CD103^+^ DCs were required for IL-17-producing ILCs responses. Contrasting reports indicate that, relative to WT mice, *L*. *major* infection of *Batf3*^*-/-*^ mice develop either exacerbated [25] or similar pathology [26]. In our study, we did not observe differences in IFN-γ production from αβ T cells or parasite load in *Batf3*^*-/-*^ mice compared to wild-type mice early after infection. However, we did find that colonization with *S*. *epidermidis* in *Batf3*^*-/-*^ mice failed to induce IL-17 from RORγt^+^ ILCs or exacerbate the pathology in *L*. *major* infected mice. These results are consistent with reports showing a role for CD103^+^ DCs in priming innate immune cells for IL-17 production. For example, the expansion of CD8^+^ T cells producing IL-17 following colonization of mice with a *S*. *epidermidis* strain was dependent on CD103^+^ DCs [21]. Similarly, the bacterial component flagellin induces IL-23 production by intestinal CD103^+^CD11b^+^ DCs to activate RORγt^+^ ILC3s [40], and microbiota-activated CD103^+^ DCs drive γδT17 proliferation and activation [41]. In parallel, we found WT but not *Batf3*^*-/-*^ mice, which lack CD103^+^ DCs, express transcripts for IL-23. IL-17A^+^ ILCs express IL-23R and blocking IL-23 abrogated the inflammatory responses and IL-17 production from ILCs in colonized mice. Thus, our data show that there is a requirement for CD103^+^ DCs for IL-23 but it does not exclude a contributing role for other DCs in the skin [21].

Together our data support the notion that microbiota-driven IL-17^+^RORγt^+^ILC activation can promote increased immunopathology, thus further demonstrating the role that IL-17 plays in cutaneous leishmaniasis. In patients these responses may be further augmented by bacteria deposited in the skin when sand flies take a blood meal [42]. The importance of IL-17 in promoting disease may not be confined to murine models, as patients with classical cutaneous leishmaniasis and mucosal leishmaniasis express IL-17 in lesions [30,43]. Similarly, leishmania infection induces alterations to the skin microbiome in patients as well as mice [9,31]. Accordingly, we found *L*. *braziliensis* patients often had a dominant *Staphylococcus* dysbiosis, which could be a driver of increased IL-17 [9]. Taken together, these results provide a rationale for therapeutic targeting of commensals, innate lymphocytes, and IL-17 for the treatment of cutaneous leishmaniasis.

## Methods

### Ethics statement

All animal studies were performed in strict accordance with the recommendations in the Guide for the Care and Use of Laboratory Animals of the National Institutes of Health, and the guidelines of the University of Pennsylvania Institutional Animal Use and Care Committee. The protocol was approved by the Institutional Animal Care and Use Committee, University of Pennsylvania Animal Welfare Assurance Number D16-00045 (A3079-01).

### Mice

Male wild-type C57BL/6 mice were purchased from Charles River Laboratories (Durham, NC). Rag1^−/−^ (B6.129S7-RAG1^tm1Mom^/J) mice were purchased from The Jackson Laboratory and bred in our facility. *Batf3*^*-/-*^ (B6.129P2(C)-Batf3^tm1Kmm^/J) mice were the gift from Drs. C. Hunter and D. Herbert (University of Pennsylvania, PA.) All mice were maintained in specific pathogen-free facilities at the University of Pennsylvania. Mice were randomly assigned to experimental groups and were 6–8 wk old at the start of the experiment and were age-matched within each experiment.

### *L*. *major* culture and infection

*L*. *major* (WHO/MHOM/IL/80/Friedlin) parasites were grown in Schneider’s Drosophila medium (GIBCO BRL, Grand Island, NY, USA) supplemented with 20% heat-inactivated fetal bovine serum (FBS) (Invitrogen USA), and 2 mML-glutamine for 4–5 days. The infectious metacyclic promastigotes of *L*. *major* were isolated by Ficoll (Sigma) density gradient centrifugation [44]. Mice were infected intradermally in the ear with 1x10^6^ *L*. *major* parasites.

### Lesion measurement and pathology score

The development of lesions was monitored weekly by measuring the ear thickness with a digital caliper (Fisher Scientific). Ear swelling/thickness was determined for individual mice by subtracting the ear thickness before treatment from that after treatment at the different time points. Inflammation and pathology were assessed by using the following inflammatory features: swelling/redness, deformation, ulceration, and loss of tissue. Based on the macroscopic appearance of the skin each feature was scored as no symptom (0), mild (1), moderate (2) and severe (3) of individual mice. The scores were summed, resulting in a maximal score of 12. Parasite burden in lesion tissues was assessed using a limiting dilution assay as previously described [9].

### *S*. *epidermidis* and *S*. *xylosus* colonization

*Staphylococcus epidermidis* strain Tu3298 expressing a fluorescent protein mCherry was a gift of Dr. Tiffany Scharschmidt (UCSF) [45] and *Staphylococcus xylosus* an isolate that was cultured from the ears of *L*. *major* infected mice [9]. For topical association, the bacteria were cultured for 24 hours in a shaking incubator, washed and re-suspended in PBS. 10^8^−10^9^ CFUs of bacteria were applied to the back and ears of the mouse using sterile cotton swabs, every day for a total of 5 days before injecting the *L*. *major* to create dysbiotic mice. For CFU quantification digested ears were plated on soy agar plate and incubated overnight at 37°C.

### Depletion of ILCs and neutrophils

To deplete ILCs, *Rag1*^*-/-*^ mice were injected with 100 μg anti-CD90.2 antibody (30H12; BioXCell). To deplete neutrophils WT mice were injected with 200 μg anti-Ly6G antibody (1A8; BioXCell). In both cases mice were injected with the antibody one day before the start of the *S*. *epi* colonization at day -5 and before the *L*. *major* injection at day -1 and then at day 5 and at day 10.

### Neutralization of IL-17A and IL-23

To study the effect of IL-17A and IL-23 in mediating the immunopathology, Rag1^-/-^ or WT mice were injected with 500 μg anti-IL-17A antibody (17F3; BioXCell) or anti-IL-23 antibody (G23-8; BioXCell). In both cases mice were injected with the antibody one day before the start of the *S*. *epi* colonization at day -5 and before the *L*. *major* injection at day -1 and then at day 5 and at day 10.

### RNA isolation and qRT-PCR with reverse transcription

RNeasy Fibrous Tissue Mini Kit (Qiagen, Hilden, Germany) was used to extract the RNA from whole ears according to the manufacturer’s instructions. For gene expression analysis, real-time PCR was performed using SuperScript One Step RT–PCR (PCR with reverse transcription) kit (Invitrogen). Primers and probes (FAM labelled) were purchased from Applied Biosystems/Thermo Fisher Scientific (Foster City, CA, USA) to quantify mRNAs. The following genes were used for analysis. *Gapdh (catalog number; Mm99999915_g1)*, *Il1a (Mm00439620_m1)*, *Il1b (Mm00434228_m1)*, *Il12a (Mm00434169_m1)*, *Il23a (Mm00518984_m1)*, *Il33 (Mm00505403_m1)*. The reactions were run on an Applied Biosystems ViiA 7 Real-Time PCR system using the standard protocol provided by Invitrogen. The expression of mRNAs was normalized to *Gapdh* mRNA by calculating 2^−ΔCt^. The threshold cycle at the limit of detection for cytokine mRNAs was 40, and when mRNAs were undetectable by RT–PCR these cycle numbers were used for calculating 2^−ΔCt^.

### Flow cytometry analysis

To obtain single cell suspensions for flow cytometry, ventral and dorsal sheets of the ear were separated from the cartilage and incubated for 90 min at 37°C in RPMI 1640 (Invitrogen, Grand Island, NY, USA) containing 0.01% DNAse (Sigma-Aldrich) and 0.25 mg ml^−1^ Liberase (Roche Diagnostics, Chicago, IL, USA). The digested ears were passed through a 1 ml syringe to make single-cell suspensions. The cells were filtered through 70 μm nylon mesh and washed before activation and/or staining. The following antibodies were used at 1:100 dilutions according to the manufacture’s specifications. CD45 (30-F11, eBiosciences), CD3 (17A2, eBiosciences), CD11b (M1/70, eBiosciences), CD19 (eBioID3, eBiosciences), NK.1.1 (PK136, eBiosciences), TER-119 (Ter-119, eBiosciences), FceR1 (MAR1, eBiosciences), βTCR (H57-597, eBiosciences), IL-1R (129304, R&D Systems), IL-12R (2.4E6, BD Pharmingen), IL-23R (12B2B64, Biolegend), IL-33R (RMST2-2, eBiosciences), Ki67 (SolA15, eBiosciences), Ly6G (1A8, eBiosciences), Ly6C (AL-21, BD Pharmingen), RoRγt (B2D, eBiosciences), IL-17A (TC11-18H10, BD Pharmingen), IL-13 (eBio13A, eBiosciences), IL-5 (TFRK5, Invitrogen), and IFN-γ (XMG 1–2, eBiosciences). For intracellular cytokine staining cells were incubated for 4 hours with Leukocyte activating cocktail (BD Biosciences) in DMEM containing 2 mM L-glutamine (Invitrogen), and following surface staining cells were fixed and permeabilized according to the manufacturer’s instructions using the BD Cytofix/Cytoperm Plus Kit (BD. For counting the cells AccuCount Fluorecent particles (Spherotech, Lake Forest, IL, USA) were used. The stained cells were run on an LSR-II flow cytometer (BD Biosciences, San Jose, CA, USA) and the acquired data were analyzed using FlowJo software (Tree Star, Ashland, OR, USA).

### *L*. *major* antigen stimulation and IFN-γ ELISA

Leishmanial antigen (Ag) was obtained from stationary-phase promastigotes of *L*. *major* by resuspending parasites at 10^9^ parasites/ml in PBS and conducting 20 freeze/thaw cycles. For measurements of Ag-specific IFN-γ production, the infected skin draining lymph node was removed, mechanically dissociated, and single-cell suspensions were prepared. Cells were resuspended in complete RPMI (Life Technologies) supplemented with 10% heat-inactivated FBS (Life Technologies), 2 mML-glutamine (Sigma), 100 U penicillin, and 100 mg streptomycin (Sigma) per milliliter and 0.05 mM 2-ME (Sigma). Cells were plated at 4 x 10^6^ cells/ml in 1 ml in 48-well plates and incubated at 37°C in 5% CO2 with 20 x 10^6^ *L*. *major* parasites/ml. Supernatants were collected after 72 h and stored at -20°C until they were assayed by sandwich ELISA using paired mAb to detect IFN-g (eBioscience).

### Statistical analysis

Mice were randomly assigned to the treatment groups and number of mice per group used in an experiment is depicted in the corresponding figure legend. Two-tailed unpaired Student’s t-test with Welch’s correction or one-way ANOVA with Tukey’s multiple comparisons were performed for significance. Mean is represented as standard error of mean (SEM) or standard deviation (SD) as shown in each figure legends. For comparing same samples/groups at different time points SD was used to represent the mean. p value < 0.05 is considered as significant.

### Supplemental material

Supplemental figures can be found in the online version of this article.

## Supporting information

S1 Fig Early IFN-γ and IL-13 induction in ILCs after *L. major* infection. **(A,B)** Percent and number of IFN-γ^+^ ILCs from ILCs in None (uninfected) and *L*. *major* infected mice at week one. **(C,D)** Percent and number of IL-13^+^ ILCs from ILCs in None (uninfected) and *L*. *major* infected mice at week one. Cells were stimulated with Pma/Ino for 4 hours. Data are from two experiments with a total of five to eight mice in each group (B,D). Numbers within the flow plot show percent of cells within the gated box. Error bars shows SEM. ns, not significant. Two-tailed unpaired Student’s t-test with Welch’s correction.
(TIF)

S2 Fig Early IFN-γ induction in T cells after *L*. *major* infection. **(A,B)** Percent and number of IFN-γ^+^ γδ^low^ T cells in None (uninfected) and *L*. *major* infected mice at week one. **(C,D)** Percent and number of IFN-γ^+^ αβ T cells in None (uninfected) and *L*. *major* infected mice at week one. Cells were stimulated with Pma/Ino for 4 hours. Data are from two experiments with a total of five to six mice in each group (B,D). Numbers within the flow plot show percent of cells in within the gated box. Error bars shows SEM. ns, not significant. Two-tailed unpaired Student’s t-test with Welch’s correction.
(TIF)

S3 Fig IL-17A^+^ ILCs at the peak of inflammation. **(A)** Experimental model for *S*. *epi* colonization and *L*. *major* infection. **(B)** Skin thickness measurement during the course of infection. **(C)** Percent and number of IL-17A^+^ ILCs in different treatment groups at week 5. Cells were stimulated with Pma/Ino for 4 hours. Data are from one experiment with a total of four to eight mice in each group (B,C,D). Number within the flow plot show percent of IL-17A^+^ cells with SEM. Error bars shows SD (B) and SEM (D). Two-tailed unpaired Student’s t-test with Welch’s correction. ns, not significant, *p<0.05, **p<0.01.
(TIF)

S4 Fig Number of total ILCs and Ki67^+^ ILCs. **(A)** Number of ILCs in WT and *Batf3*^*-/-*^ mice at week two in different treatment groups. **(B)** Number of Ki67^+^ ILCs at week two in WT mice in different treatment groups. Data are from one experiment with a total of five to six mice in each group (A,B). Error bars show SEM. Two-tailed unpaired Student’s t-test with Welch’s correction. ns, not significant, *p<0.05.
(TIF)

S5 Fig mRNA analysis of IL-12a and IL-33. **(A,B)** mRNA analysis of IL-12a and IL-33 at week two in different treatment groups. Data are from one experiment with a total of five to six mice in each group (A,B). Error bars show SEM. Two-tailed unpaired Student’s t-test with Welch’s correction. ns, not significant.
(TIF)

S6 Fig Analysis of IL-33R, IL-1R and IL-12R on IL-17A^+^ and IL-17A^-^ ILCs. Flow cytometry analysis of IL-33R, IL-1R and IL-12R on IL-17A^+^ and IL-17A^-^ ILCs in the *S*. *epi* colonized and *L*. *major* infected mice at week two. Total ILCs were used as isotype control. Data are from two experiments with a total of three to four mice in each group. Error bars show SEM. Two-tailed unpaired Student’s t-test with Welch’s correction. ns, not significant ***p<0.001.
(TIF)

S7 Fig IFN-γ and IL-5 in *Rag1^-/-^* mice. **(A)** Representative flow cytometry plot of IFN-γ and IL-5 staining from ILCs in *Rag1*^*-/-*^ mice treated with *S*. *epi*. *or L*. major alone or co-treated with *S*. *epi and L*. *major*. Numbers represent percent of cells within the gated box. **(B)** Number of IFN-γ and IL-5 producing ILCs in *Rag1*^*-/-*^ mice treated with *S*. *epi*. *or L*. *major alone* or co-treated with *S*. *epi and L*. *major*. Cells were stimulated with Pma/Ino for 4 hours. Number within the flow plot shows percent of cells within the gated box. Error bars shows SEM. Data are from one or two experiments with a total of three to six mice in each group.
(TIF)

S8 Fig Immunopathology in WT and *Rag1^-/-^* mice. **(A)** Experimental model for *S*. *epi* colonization and *L*. *major* infection in WT and *Rag1*^*-/-*^ mice. **(B,C)** Skin thickness and pathology score during the course of infection. Data are from one experiment with a total of three to five mice in each group. Error bars show SEM. Two-tailed unpaired Student’s t-test with Welch’s correction. ns, not significant, *p<0.05.
(TIF)
