# Supplementary figures and images for: Microbiota instruct IL-17A-producing innate lymphoid cells to promote skin inflammation in cutaneous leishmaniasis
Source: PLoS Pathog. 2021 Oct 26;17(10):e1009693. doi: 10.1371/journal.ppat.1009693 (PMC8570469; doi:10.1371/journal.ppat.1009693)

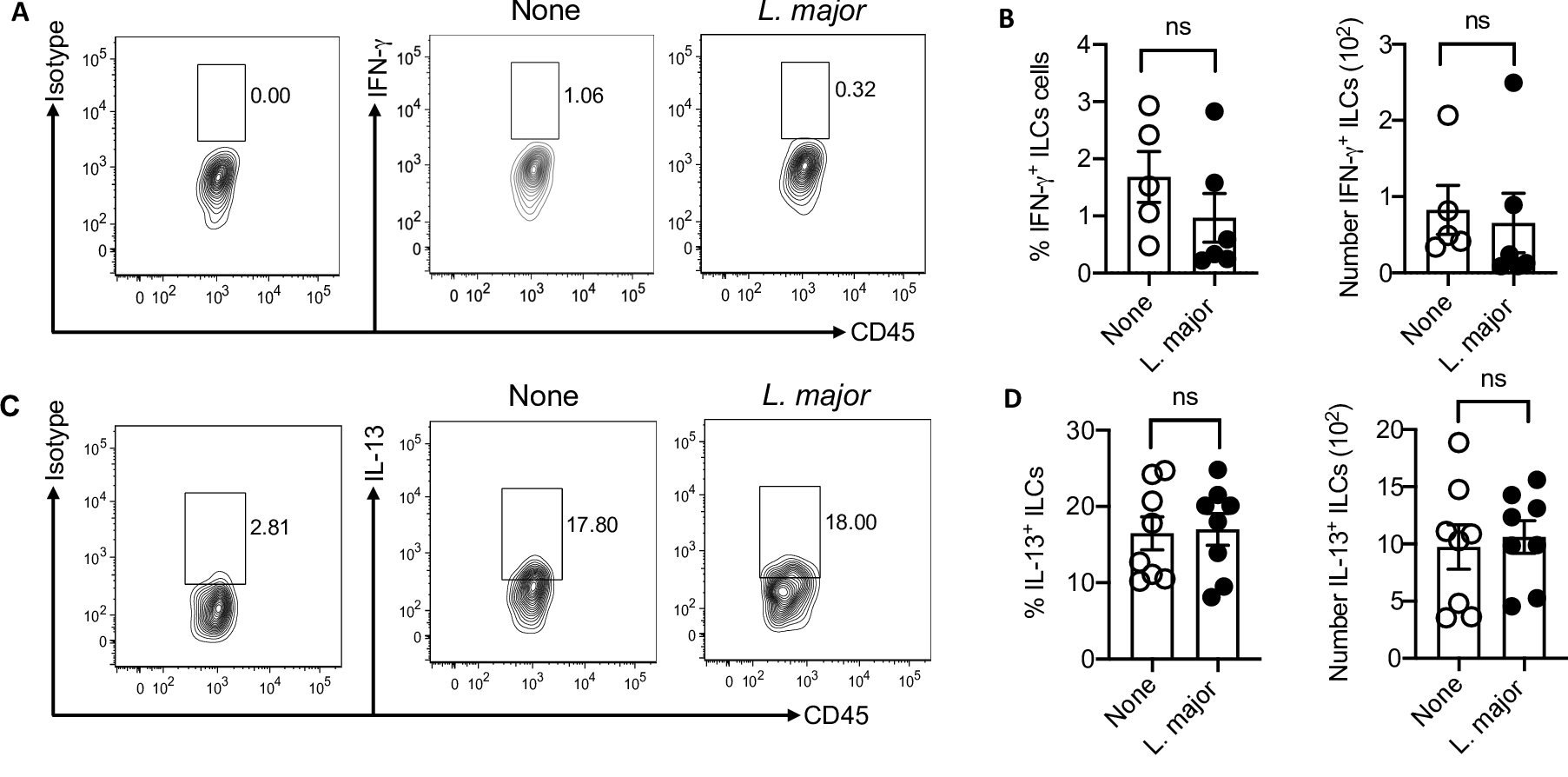

Supplement: S1 Fig — (A,B) Percent and number of IFN-γ+ ILCs from ILCs in None (uninfected) and L. major infected mice at week one. (C,D) Percent and number of IL-13+ ILCs from ILCs in None (uninfected) and L. major infected mice at week one. Cells were stimulated with Pma/Ino for 4 hours. Data are from two experiments with a total of five to eight mice in each group (B,D). Numbers within the flow plot show percent of cells within the gated box. Error bars shows SEM. ns, not significant. Two-tailed unpaired Student’s t-test with Welch’s correction. (TIF) [file ppat.1009693.s001.tif]

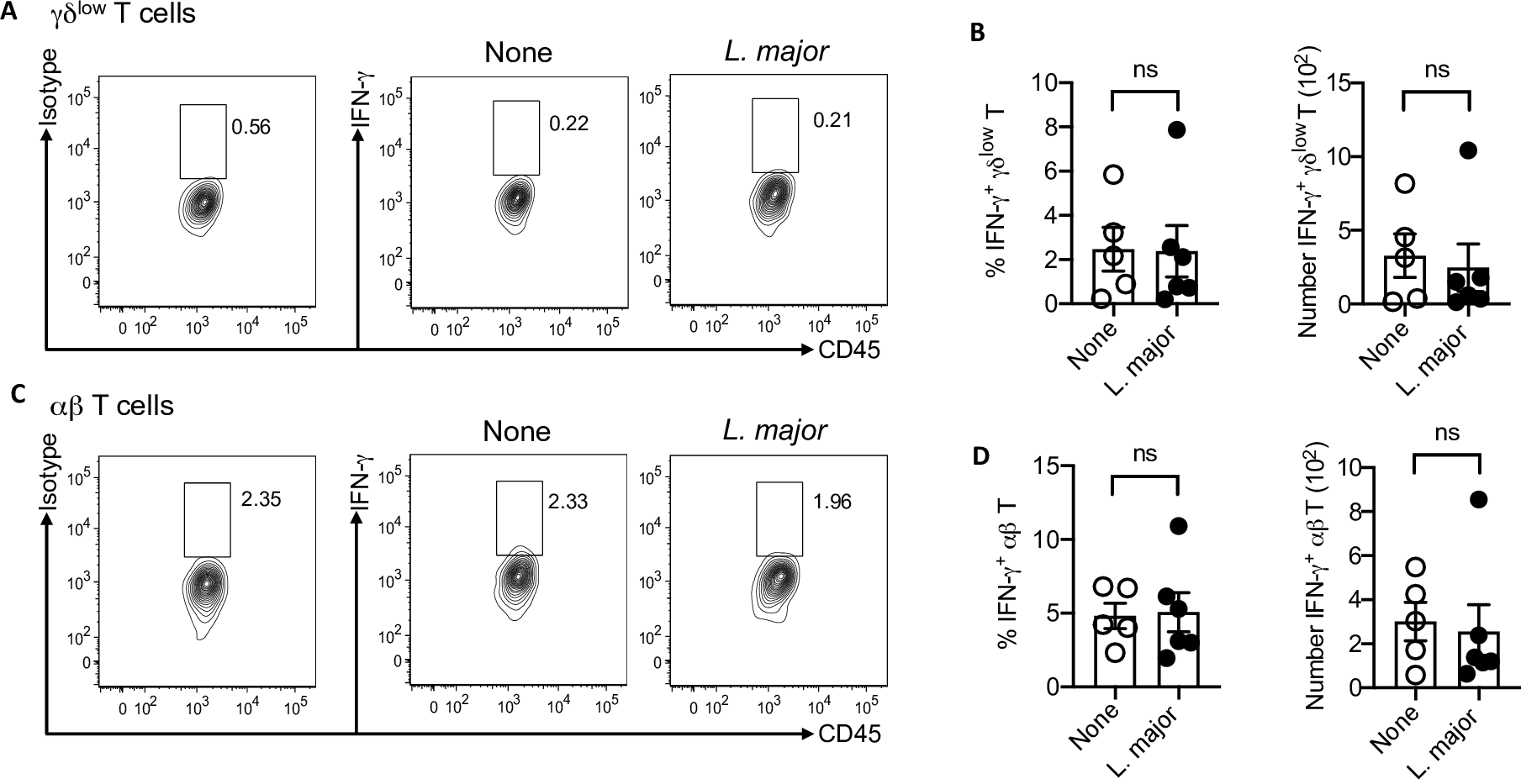

Supplement: S2 Fig — (A,B) Percent and number of IFN-γ+ γδlow T cells in None (uninfected) and L. major infected mice at week one. (C,D) Percent and number of IFN-γ+ αβ T cells in None (uninfected) and L. major infected mice at week one. Cells were stimulated with Pma/Ino for 4 hours. Data are from two experiments with a total of five to six mice in each group (B,D). Numbers within the flow plot show percent of cells in within the gated box. Error bars shows SEM. ns, not significant. Two-tailed unpaired Student’s t-test with Welch’s correction. (TIF) [file ppat.1009693.s002.tif]

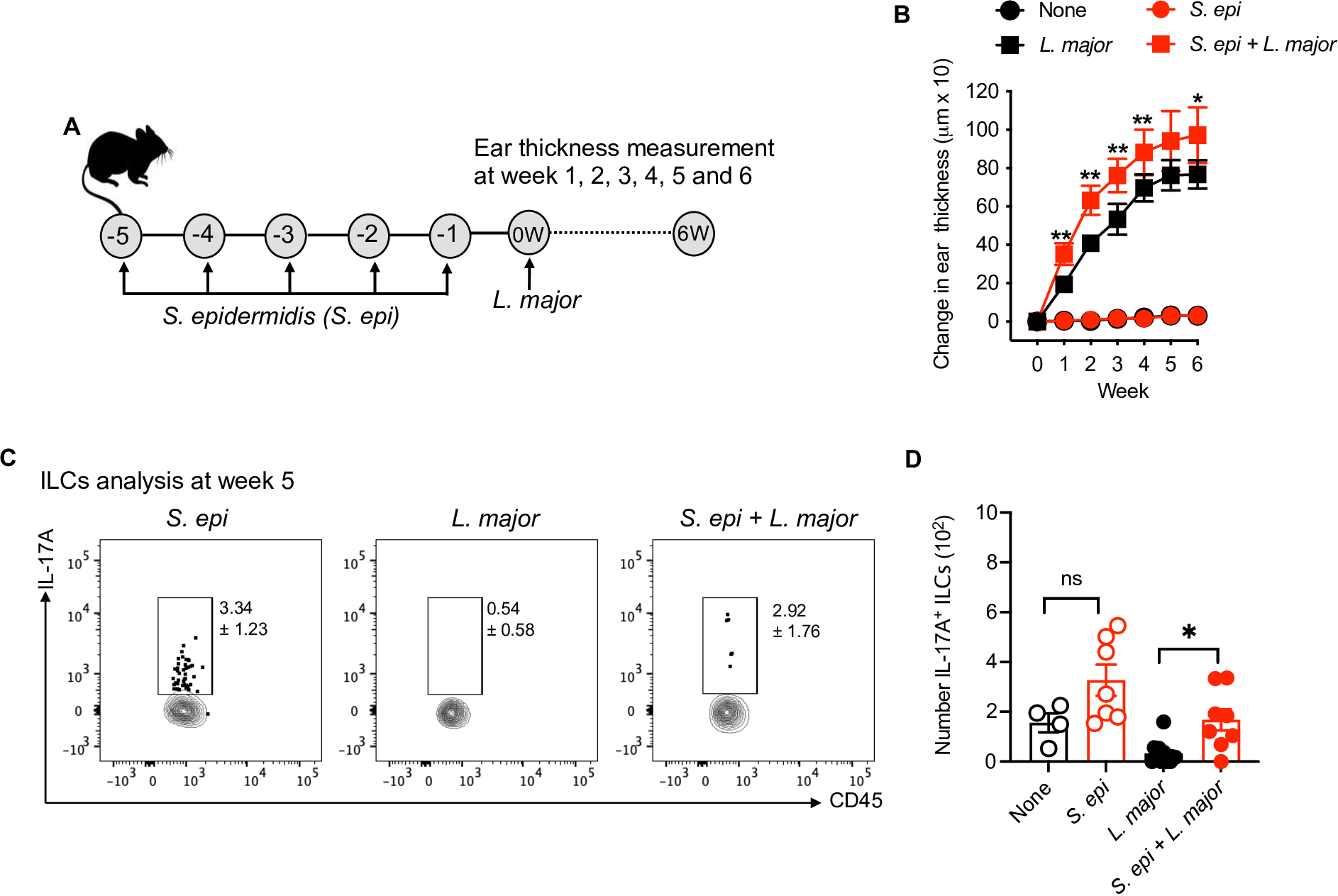

Supplement: S3 Fig — (A) Experimental model for S. epi colonization and L. major infection. (B) Skin thickness measurement during the course of infection. (C) Percent and number of IL-17A+ ILCs in different treatment groups at week 5. Cells were stimulated with Pma/Ino for 4 hours. Data are from one experiment with a total of four to eight mice in each group (B,C,D). Number within the flow plot show percent of IL-17A+ cells with SEM. Error bars shows SD (B) and SEM (D). Two-tailed unpaired Student’s t-test with Welch’s correction. ns, not significant, *p<0.05, **p<0.01. (TIF) [file ppat.1009693.s003.tif]

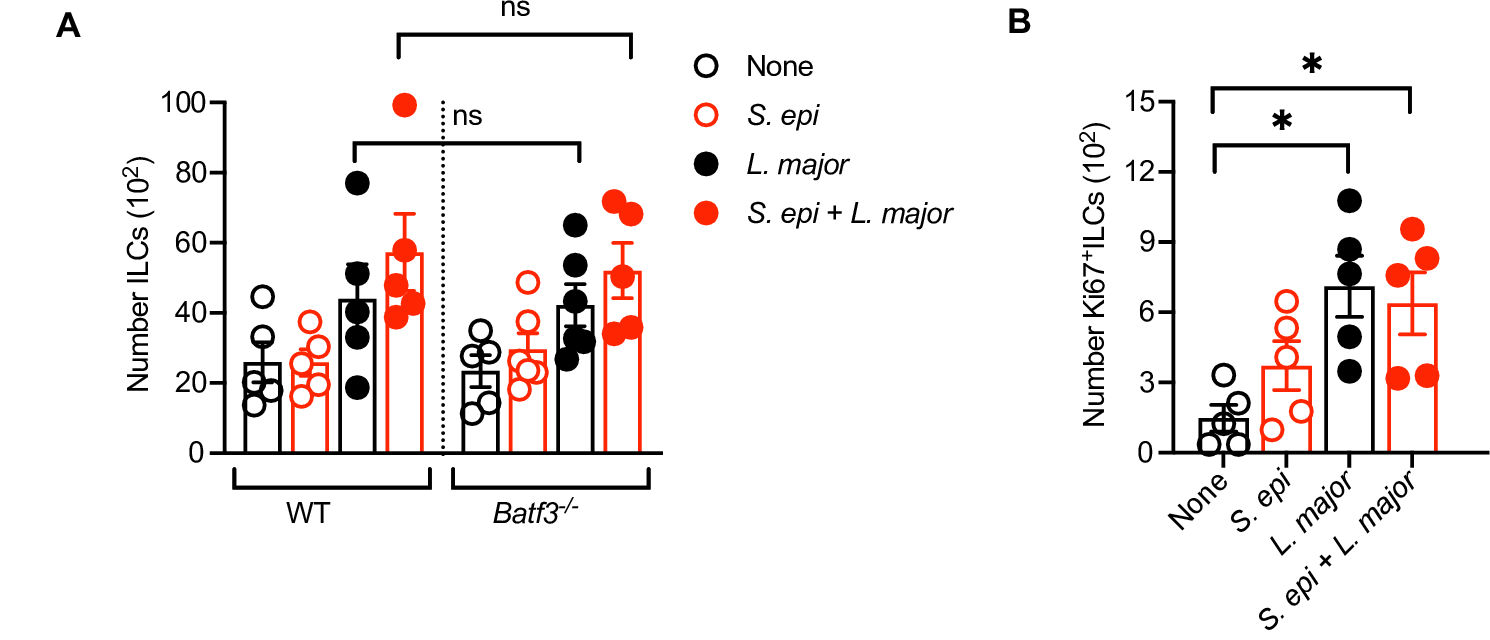

Supplement: S4 Fig — (A) Number of ILCs in WT and Batf3-/- mice at week two in different treatment groups. (B) Number of Ki67+ ILCs at week two in WT mice in different treatment groups. Data are from one experiment with a total of five to six mice in each group (A,B). Error bars show SEM. Two-tailed unpaired Student’s t-test with Welch’s correction. ns, not significant, *p<0.05. (TIF) [file ppat.1009693.s004.tif]

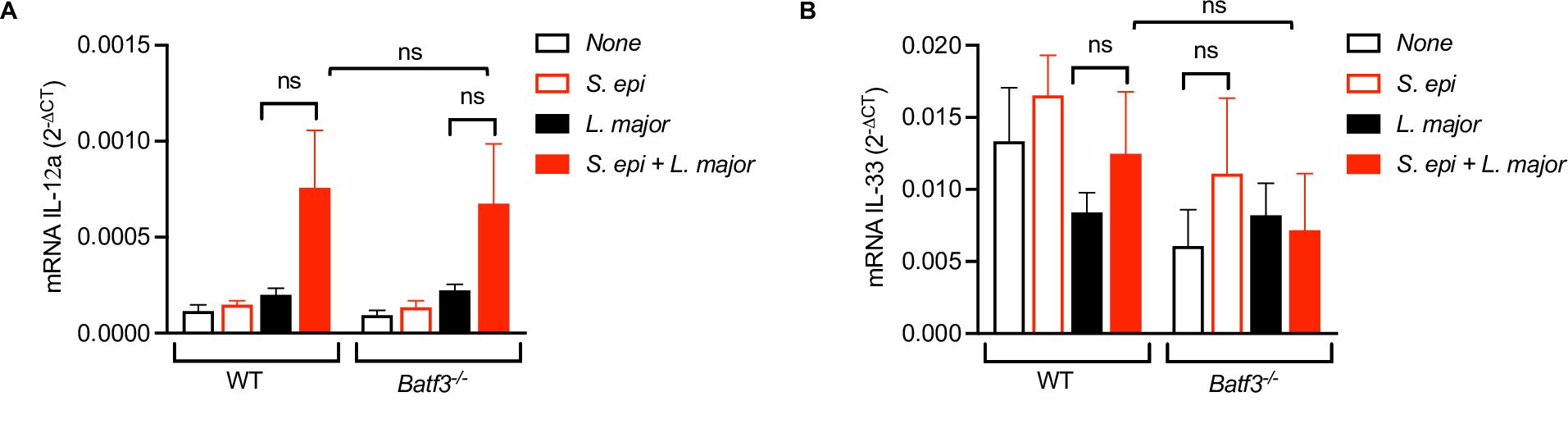

Supplement: S5 Fig — (A,B) mRNA analysis of IL-12a and IL-33 at week two in different treatment groups. Data are from one experiment with a total of five to six mice in each group (A,B). Error bars show SEM. Two-tailed unpaired Student’s t-test with Welch’s correction. ns, not significant. (TIF) [file ppat.1009693.s005.tif]

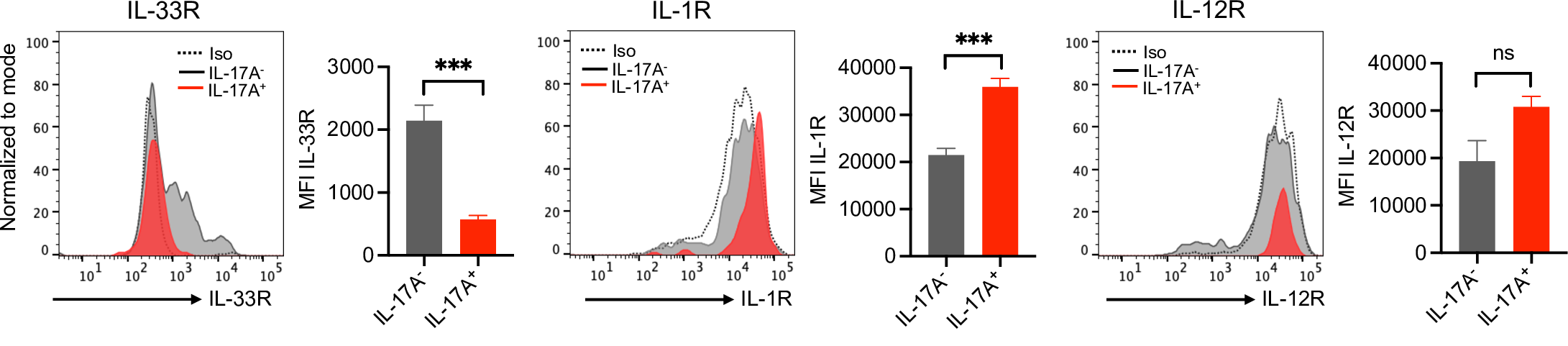

Supplement: S6 Fig — Flow cytometry analysis of IL-33R, IL-1R and IL-12R on IL-17A+ and IL-17A- ILCs in the S. epi colonized and L. major infected mice at week two. Total ILCs were used as isotype control. Data are from two experiments with a total of three to four mice in each group. Error bars show SEM. Two-tailed unpaired Student’s t-test with Welch’s correction. ns, not significant ***p<0.001. (TIF) [file ppat.1009693.s006.tif]

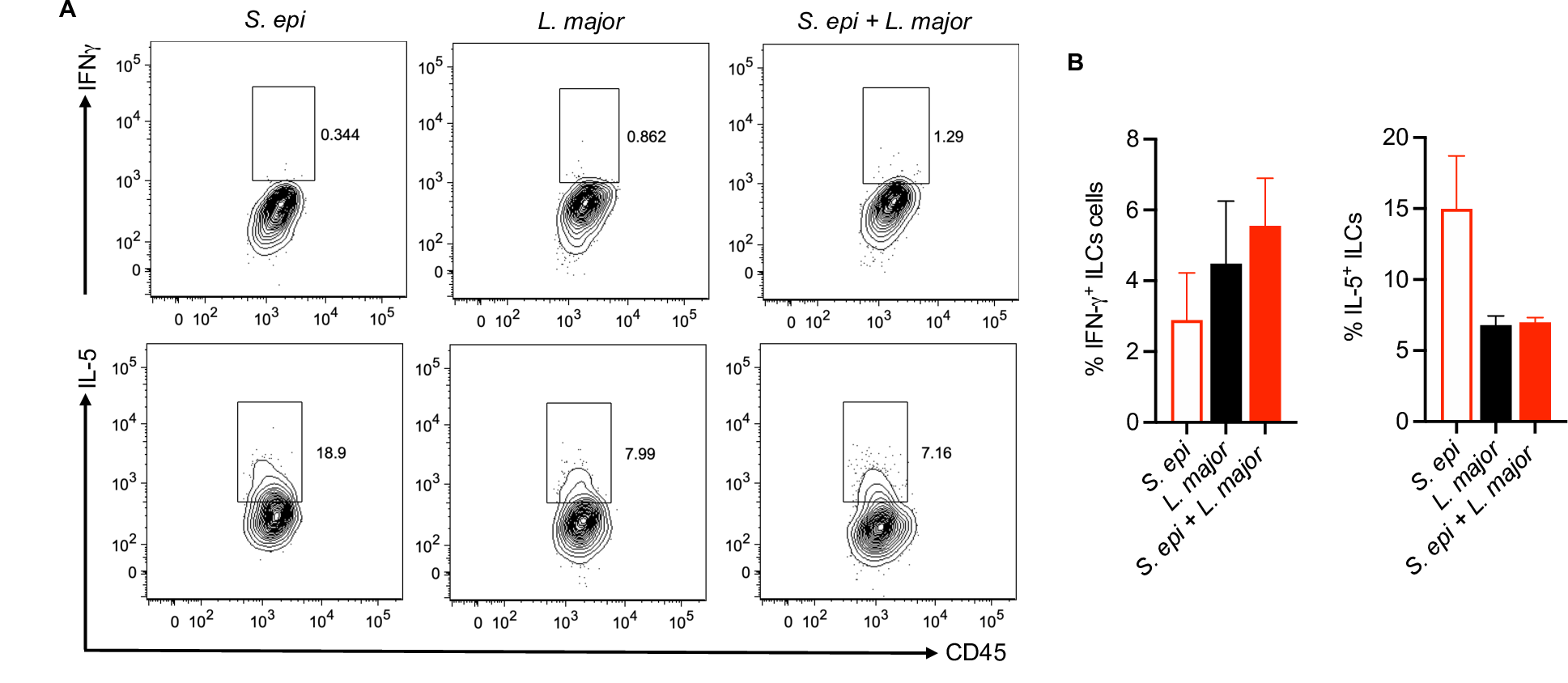

Supplement: S7 Fig — (A) Representative flow cytometry plot of IFN-γ and IL-5 staining from ILCs in Rag1-/- mice treated with S. epi. or L. major alone or co-treated with S. epi and L. major. Numbers represent percent of cells within the gated box. (B) Number of IFN-γ and IL-5 producing ILCs in Rag1-/- mice treated with S. epi. or L. major alone or co-treated with S. epi and L. major. Cells were stimulated with Pma/Ino for 4 hours. Number within the flow plot shows percent of cells within the gated box. Error bars shows SEM. Data are from one or two experiments with a total of three to six mice in each group. (TIF) [file ppat.1009693.s007.tif]

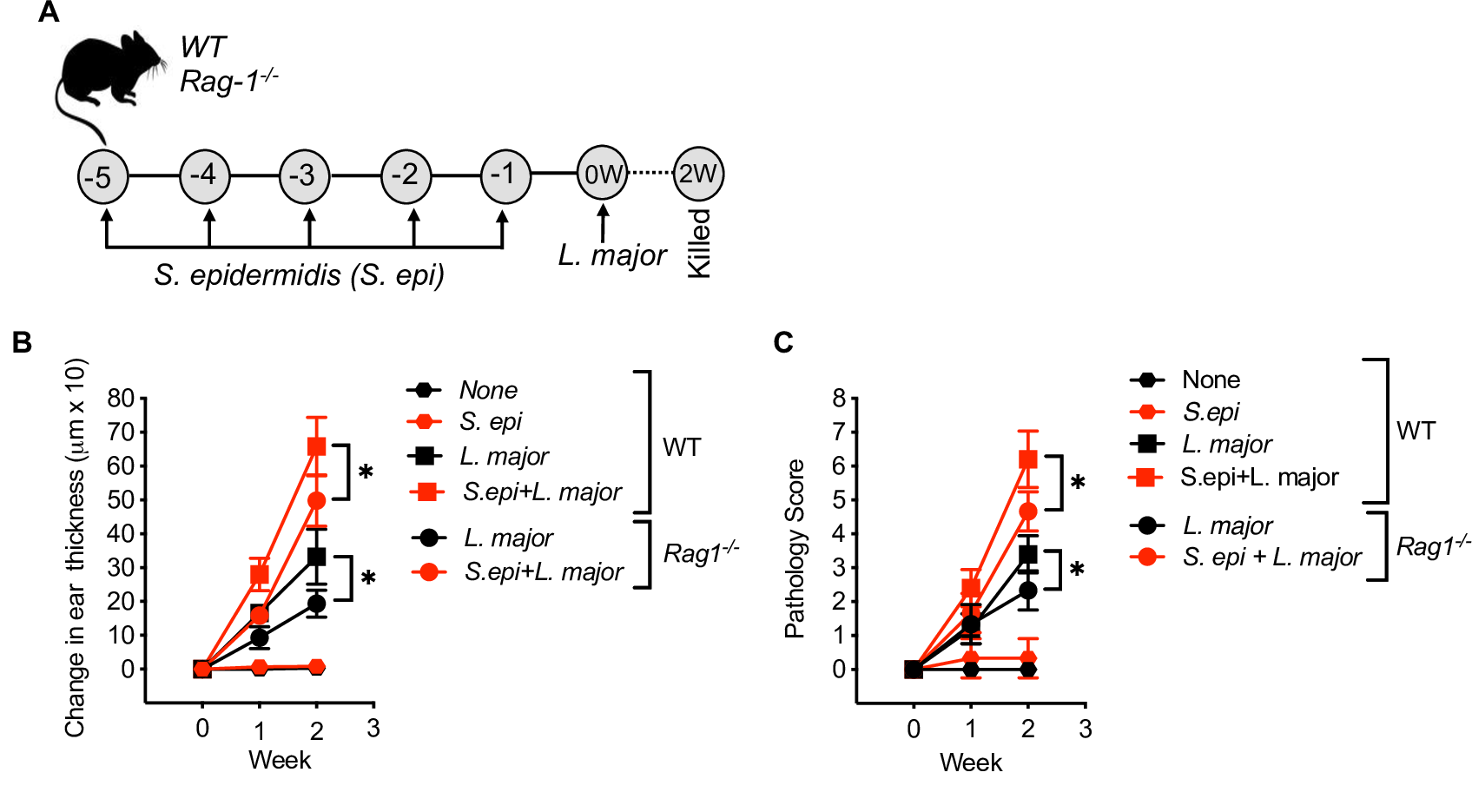

Supplement: S8 Fig — (A) Experimental model for S. epi colonization and L. major infection in WT and Rag1-/- mice. (B,C) Skin thickness and pathology score during the course of infection. Data are from one experiment with a total of three to five mice in each group. Error bars show SEM. Two-tailed unpaired Student’s t-test with Welch’s correction. ns, not significant, *p<0.05. (TIF) [file ppat.1009693.s008.tif]
